# Resistant Potato Starch Supplementation Increases Serum Antioxidant Levels in a Randomized Trial

**DOI:** 10.3390/metabo15100661

**Published:** 2025-10-10

**Authors:** Jason R. Bush, Jun Han, David R. Goodlett

**Affiliations:** 1 MSP Starch Products Inc., Carberry, MB R0K 0H0, Canada; 2 The University of Victoria-Genome British Columbia Proteomics Centre, Victoria, BC V8Z 7X8, Canada; hanjun@proteincentre.com (J.H.); goodlett@uvic.ca (D.R.G.); 3 Division of Medical Sciences, University of Victoria, Victoria, BC V8N 4V3, Canada

**Keywords:** prebiotic, potato, resistant starch, antioxidant, retinol, α-tocopherol, coenzyme Q10, hydroxykynurenine

## Abstract

**Background/Objectives**: The prebiotic effect of resistant potato starch (RPS) has been demonstrated, but the antioxidant properties associated with this ingredient have not been explored. **Methods**: We performed post hoc analysis of serum metabolomic data from a clinical trial evaluating 3.5 g RPS per day consumption (*n* = 24) versus a placebo (*n* = 24) for 4 weeks in a randomized clinical trial (NCT05242913). **Results**: Levels of the exogenous antioxidants all-trans retinol and α-tocopherol increased in the RPS-consuming group. Among endogenous antioxidants, the concentration of coenzyme Q10 (CoQ10) increased in both treatment groups, while uric acid was unaffected. Hippuric acid, a marker of polyphenol metabolism, was unaffected by treatment, as was the abundance of the tryptophan metabolites kynurenine and 3-hydroxyanthranillic acid. However, levels of 3-hydroxykynurenine were decreased in both treatment groups. Levels of the advanced glycation end products NƐ-(1-carboxymethyl)-L-lysine and NƐ-(1-carboxyethyl)-L-lysine, markers of chronically elevated oxidative stress, were unaffected by treatment. Notably, increases in serum all-trans retinol were correlated with increases in *Akkermansia*. **Conclusions**: RPS enhances the absorption of antioxidants all-trans retinol and α-tocopherol from the diet and also influences CoQ10 levels and tryptophan metabolism. Future studies assessing the physiological consequences of enhanced antioxidant absorption in people consuming RPS over a longer duration are warranted.

## 1. Introduction

Reactive oxygen species (ROS) produced by mitochondria during aerobic respiration and reactive nitrogen species (RNS) produced during immune responses can cause numerous deleterious effects, including damage to lipids, proteins, and DNA [1]. The body possesses an antioxidant system to mitigate these adverse effects, including a combination of enzymes, proteins, and metabolites. Antioxidants may be endogenously synthesized or derived from the diet. Examples of endogenous antioxidants include α-lipoic acid [2], coenzyme (Co) Q10 [3], glutathione [4], and uric acid [5]. Vitamins A, C, and E are exclusively obtained from the diet [6], while CoQ10 can be endogenously synthesized [7] or obtained from the diet [8]. Polyphenols can also produce antioxidant effects, donating hydrogen atoms to adopt a quinone status [9]. Dysregulation of the redox balance in diseased states can create an imbalance between ROS and antioxidants [10]. For example, elevated blood glucose levels in diabetics creates oxidative stress, causing lipid peroxidation, neuronal death, and neuropathy [11]. Administration of exogenous α-lipoic acid can reduce these effects and lessen neuropathy symptoms in diabetics [11].

Potato tubers are an important starchy food in most cultures [12]. While the digestible carbohydrate composition of potatoes is often viewed negatively due to the impact on blood glucose levels, potatoes are the largest contributor of vegetable phenolics and cellular antioxidant activity to the American diet [13]. Although anthocyanins are typically only abundant in dark-fleshed potatoes, white-fleshed Burbank Russet potatoes contain carotenoids with substantial hydrophilic and lipophilic oxygen radical absorbance capacity values [14]. Reflecting this health-promoting property, Health Canada includes dry or fresh tuber as a source of antioxidants under the Natural Health Product Antioxidants Monograph [15]. Natural health products containing this material can obtain a pre-cleared “source of antioxidants” claim that can be advertised in the Canadian retail marketplace. Despite these properties, few studies examining the antioxidant effects of potato products in humans have been performed.

Potatoes contain other important nutrients that could likewise influence antioxidant activities. For example, uncooked potatoes also contain the highest natural source of resistant starch (RS) by weight [16]. Resistant starch is defined as ‘the sum of starch and starch-degradation products that, on average, reach the human large intestine’ [17]. In addition to providing a source of dietary fiber, resistant potato starch (RPS) has important prebiotic properties, including stimulating the growth of *Bifidobacterium* [18,19,20] and *Akkermansia* [20] in the gut, improving stool form [20], enhancing intestinal barrier function [21], improving markers of insulin resistance [22,23], and promoting healthy choline and phospholipid metabolism [24]. However, studies evaluating RPS in vitro, in animal models and in humans, have not previously reported antioxidant effects. If RPS were shown to have antioxidant-promoting properties, it would broaden the number of benefits associated with RPS supplementation.

Studies of ingredients that modulate the composition or activity of the gut microbiome have reported antioxidant effects. For example, probiotic administration generally has beneficial effects on markers of oxidative stress in type 2 diabetics, but beneficial effects of prebiotics were not reported [25]. Prebiotics, by their nature, cannot be absorbed into the blood and pass through the stomach to the intestines, where resident microbiota utilize them to promote a health benefit [26]. For this reason, antioxidant properties due to the prebiotic’s chemical structure are likely to only influence redox reactions in the gut. Systemic antioxidant effects reported in prebiotic studies likely involve the induction of endogenous antioxidant systems and/or the absorption of exogenous antioxidants. Based on the antioxidant properties reported for potatoes [12,13,14], we hypothesized that RPS consumption would increase antioxidant capacity in humans consuming this ingredient. We therefore examined effects on endogenous and exogenous antioxidant levels using targeted serum metabolomics in people consuming RPS or placebo and correlated these effects with changes in key gut microbiota.

## 2. Materials and Methods

### 2.1. Investigational Product

The resistant potato starch (RPS) used in this study was Solnul^®^ (MSP Starch Products Inc., Carberry, MB, Canada), an unmodified RS Type 2 produced via a proprietary processing method to preserve resistant starch (RS). Solnul^®^ contains a minimum RS content of 60% (AOAC 2002.02). The placebo used was a high-amylopectin corn starch (Amioca; Ingredion, Brampton, ON, Canada) that is fully digested and has no discernible effects on the gut microbiota [27].

### 2.2. Study Design

The participants of this study have been described in detail, including the sample size calculation [20]. Importantly, this clinical trial was the first to evaluate the prebiotic effect of RS at a low dose that was comparable in size to soluble prebiotics, such as inulin, fructooligosaccharides, and galactooligosaccharides [20]. Previous studies found prebiotic benefit at doses of 30 g of RS per day or higher [19,22,27]. In brief, healthy adults aged 18–69 years with a body mass index (BMI) of 18.0 to 34.9 kg/m^2^ were recruited and enrolled in the study. Candidate participants with a BMI ≥ 35 kg/m^2^ were excluded. Candidate participants reporting a diagnosis of irritable bowel syndrome, dyspepsia, significant gastrointestinal disorders, or other major diseases were also excluded. This allowed the clinical Principal Investigator to use objective criteria such as diagnosis, medication records, and review of recent hospitalizations to determine whether candidates were included or excluded. Those enrolled agreed to not consume any vitamins, minerals, or dietary supplements from 14 days prior to the randomization visit until the study concluded. Participants were counseled to follow their habitual diet throughout the study period and no changes in dietary intake were observed [28].

### 2.3. Clinical Trial Conduct

The study occurred between 30 October 2019 and 6 January 2020 in Guelph, ON, Canada, with participants recruited from the general population in Guelph and the surrounding area. Canadian Shield Ethics Review Board (tracking number 19-10-001; Burlington, ON, Canada) approved the study protocol and the trial was registered at ClinicalTrials.gov (NCT05242913). Written informed consent was obtained from all study participants or their legally authorized representative prior to enrollment into the study, following the Declaration of Helsinki and Council for International Organizations of Medical Sciences International Ethical Guidelines and ICH Good Clinical Practice guidelines [20].

The CONSORT diagram summarizing the study design has previously been reported [20]. The study was a randomized, double-blind, placebo-controlled, parallel-armed clinical trial designed to evaluate the prebiotic effects of daily 3.5 g of RPS (containing 3.5 g of RPS and 3.5 g placebo, with 7 g total carbohydrates), 7 g of RPS, and 7 g of placebo for 4 weeks using fecal samples to estimate gut microbiome composition and bowel movement, characterized using the Bristol Stool Form Chart [20]. Sachets without marks identifying the nature of the investigational product were provided to participants and clinic staff were blinded to the investigational product identity. Forty-eight participants completed the study protocol for the 3.5 g RPS (*n* = 24) and placebo (*n* = 24) arms. Non-fasting serum samples were collected at baseline and 4 weeks of supplementation and analyzed by targeted metabolomics to quantify circulating levels of polar metabolites and lipophilic metabolites, which were analyzed as secondary outcomes. Due to commercial interest in the effects of low RPS doses, only the impacts of 3.5 g RPS and placebo on antioxidants are reported.

### 2.4. Metabolomic Analysis

Polar metabolite analysis, including measurement of acetate, has been described in detail [21,23]. For the analysis of lipophilic CoQ10 and fat-soluble vitamins, an assay by liquid chromatography coupled to tandem mass spectrometry (LC-MS/MS) was applied. In brief, ten serially diluted, mixed calibration solutions were prepared with the use of standard substances of the measured compounds in an internal standard (IS) solution containing CoQ10-d9, all-trans retinol-d5, and α-tocopherol-d6. The IS solution was prepared in methanol–chloroform (1:1, *v*/*v*), containing butylated hydroxytoluene (100 µg/mL) as an antioxidant. The concentrations of each compound in the calibration solutions ranged from 0.01 nM to 10 µM. For sample preparation, 50 µL of serum from each subject, which had been stored at −80 °C, was thawed on ice and aliquoted into a 1.5 mL Eppendorf tube and then mixed with 50 µL of the IS solution, followed by addition of 400 µL of methanol–chloroform (3:1, *v*/*v*). The mixtures were vortexed at 3000 rpm for 1 min, ultra-sonicated in an ice-water bath for 2 min, and subsequently centrifuged at 21,000× *g* and 5 °C for 10 min in an Eppendorf 5425R centrifuge (Eppendorf Canada, Mississauga, ON, Canada). The clear supernatants were collected into 1 mL micro-vials and dried down under a gentle nitrogen gas flow at room temperature. The dried residues were dissolved in 50 µL of methanol–chloroform (1:1, *v*/*v*). Next, 6 µL aliquots of the sample solutions and the calibration solutions were injected into an Agilent Eclipse C8 (2.1 × 50 mm, 1.8 µm) column to run LC-MS/MS on a system of an Agilent 1290 Infinity II UHPLC instrument hyphenated via a Jet Stream electrospray ion source to an Agilent 6495B triple-quadrupole mass spectrometer (Agilent Technologies, Santa Clara, CA, USA). The mass spectrometer was operated in the positive-ion and dynamic multiple-reaction monitoring mode. For chromatographic separations, a binary-solvent mobile phase composed of 0.05% formic acid in water (A) and 0.05% formic acid in acetonitrile–isopropanol (1:1, *v*/*v*) (B) was used for gradient elution at 0.36 mL/min and 55 °C. The elution gradient was as follows: 0–7.5 min, 5% to 60% B; 7.5–20 min, 60% to 100% B and 20–22 min, 100% B. The column was re-equilibrated at 5% B for 3 min between injections. For quality control (QC), aliquots of 50 µL serum were pooled from 30 randomly chosen samples. In total, 50 µL aliquots of the pooled serum sample were prepared along with the batch serum samples in the same way. The resultant QC solutions were injected periodically at the beginning, in the middle and at the end of the LC-MS/MS batch runs to monitor the analytical variations. The LC-MS/MS data were recorded and processed using the Agilent MassHunter 10.0 software suite (Agilent Technologies, Santa Clara, CA, USA). Internal standard calibrated linear-regression curves of individual compounds were constructed with the data acquired from the calibration solutions, within an appropriate concentration range for each compound. Concentrations of the compounds detected in serum were calculated by interpolating the calibration curves with the analyte-to-IS peak area ratios measured from the sample solutions.

### 2.5. Statistical Analysis

Baseline and week 4 metabolite levels were compared within groups using Student’s paired, two-tailed *t*-test. Pearson correlation analysis compared metabolite changes at week 1 and week 4 to changes in *Bifidobacterium* and *Akkermansia* at week 1 and 4 to increase statistical power [27]. Differences were considered significantly different at *p* < 0.05. All comparisons were made using Excel (Version 2409; Microsoft, Redmond, WA, USA).

## 3. Results

### 3.1. Exogenous Antioxidants

**Figure 1:**
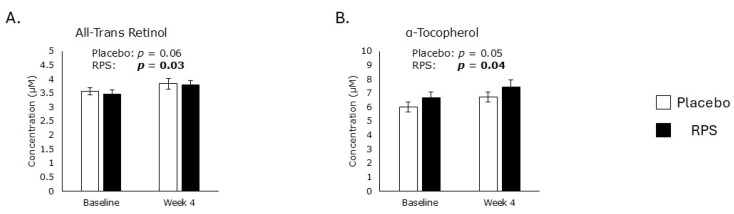
Exogenous antioxidants. RPS consumption led to increases in serum all-trans retinol levels (*p* = 0.03; (**A**)) and α-tocopherol levels (*p* = 0.04; (**B**)), while placebo treatment had no effect. Student’s *t*-test; Mean ± SEM. The *p* values of significant correlations are in bold.

Of the various exogenous antioxidants, only all-trans retinol, the major form of vitamin A, and α-tocopherol, the most abundant form of vitamin E circulating in peripheral blood, were captured in the acquired serum lipophilic metabolomics dataset. Levels of all-trans retinol increased by 9% in the RPS-consuming group after 4 weeks (*p* = 0.04) but were not affected by the placebo (*p* = 0.06; Figure 1A). Similarly, levels of α-tocopherol increased by 11% in the RPS-consuming group (*p* = 0.04) but were unaffected in the placebo group (*p* = 0.05; Figure 1B).

### 3.2. Endogenous Antioxidants

**Figure 2:**
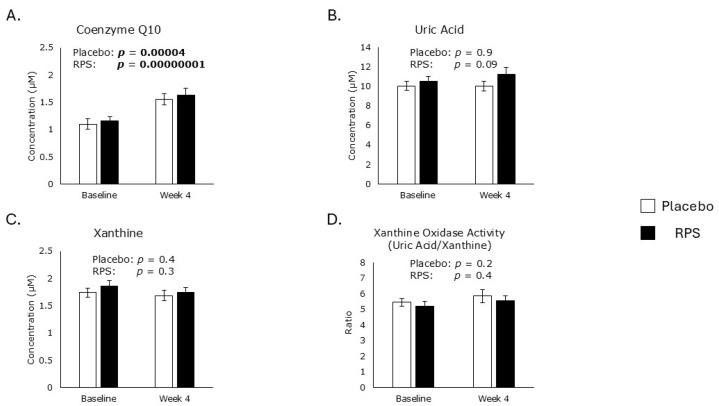
Endogenous antioxidants. Levels of coenzyme Q10 increased in both RPS (*p* = 0.00000001) and placebo (*p* = 0.00004) treatment groups (**A**), but levels of uric acid (**B**) and xanthine (**C**), and the activity of xanthine oxidase (**D**) were unaffected. Student’s *t*-test; Mean ± SEM. The *p* values of significant correlations are in bold.

Of the various circulating endogenous antioxidants, only CoQ10 and uric acid were captured in the lipophilic metabolomic dataset and the polar metabolomic datasets, respectively [21,23]. Levels of CoQ10 increased in both RPS- (40%; *p* = 0.00000001) and placebo (41%; *p* = 0.00004)-consuming groups after 4 weeks (Figure 2A). Levels of uric acid were unaffected by either treatment group (Figure 2B). Probiotic administration has been shown to influence host liver enzyme activity affecting uric acid production [29]. We therefore measured the abundance of xanthine (Figure 2C) and the activity of xanthine oxidase (Figure 2D), but neither intervention had an effect.

### 3.3. Polyphenol and Tryptophan Metabolites

**Figure 3:**
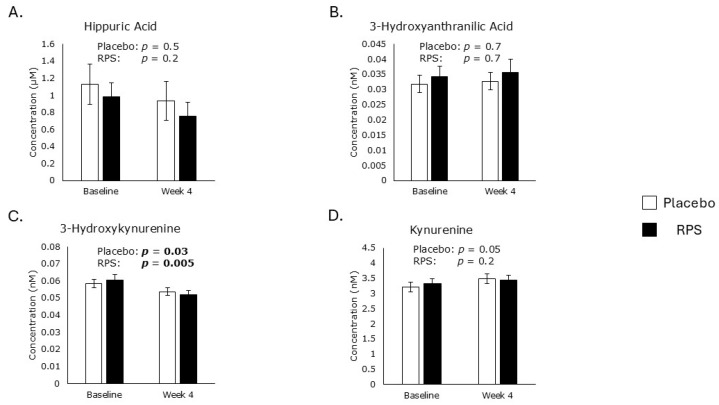
Polyphenol and tryptophan metabolites. Neither treatment affected levels of hippuric acid (**A**), 3-hydroxyanthranilic acid (**B**), or kynurenine (**D**) in serum. Both RPS (*p* = 0.005) and placebo (*p* = 0.03) treatments reduced serum levels of 3-hydroxykynurenine (**C**). Student’s *t*-test; Mean ± SEM. The *p* values of significant correlations are in bold.

Hippuric acid is a metabolite positively correlated with the intake of polyphenol-rich vegetable foods [30]. However, neither treatment influenced hippuric acid levels (Figure 3A). Tryptophan metabolites, including 3-hydroxyanthranilic acid, 3-hydroxykynurenine, and kynurenine, contain a phenolic ring and play roles in balancing physiological oxidation-reduction reactions [31]. Tryptophan levels were previously reported to be unaffected by RPS [21]. Levels of 3-hydroxykynurenine were decreased in the RPS (−14%; *p* = 0.005) and placebo (−8%; *p* = 0.03) treatment groups (Figure 3C), but neither treatment affected 3-hydroxyanthranilic acid (Figure 3B) or kynurenine levels (Figure 3D).

### 3.4. Metabolomic Markers of Oxidative Stress

**Figure 4:**
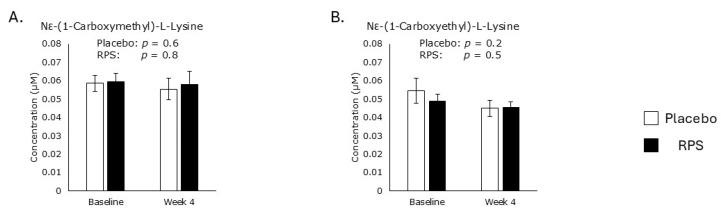
Advanced glycation end products. Neither treatment affected levels of NƐ-(1-carboxymethyl)-L-lysine (**A**) or NƐ-(1-carboxyethyl)-L-lysine (**B**) in serum. Student’s *t*-test; Mean ± SEM.

NƐ-(1-Carboxymethyl)-L-lysine and NƐ-(1-carboxyethyl)-L-lysine are produced by oxidative modification of glycated proteins during oxidative stress [32,33]. Levels of both NƐ-(1-carboxymethyl)-L-lysine (Figure 4A) and NƐ-(1-carboxyethyl)-L-lysine (Figure 4B) were unaffected by either intervention after 4 weeks of treatment.

### 3.5. Correlations Between Increases in Antioxidant Levels and Changes in Gut Microbiota

Finally, we asked whether the RPS-dependent increases in serum all-trans retinol, α-tocopherol, or CoQ10 were correlated with changes in keystone microbiome genera *Bifidobacterium* or *Akkermansia*, both of which were previously shown to increase in response to RPS supplementation [20]. For these comparisons, changes across both weeks 1 and 4 were used to increase statistical power [27]. Changes in *Bifidobacterium* were not correlated with changes in the serum levels of any of these antioxidants (Table 1). However, increases in *Akkermansia* were correlated with increases in all-trans retinol (*p* = 0.008) and there was a tendency towards a correlation between this genus and increases in α-tocopherol (*p* = 0.1; Table 1). Changes in other metabolites reported in this manuscript were not correlated with changes in *Bifidobacterium* or *Akkermansia* in participants consuming RPS (Table 1). Increases in CoQ10 were inversely correlated with changes in *Bifidobacterium* in the placebo group (*p* = 0.02; Table 2), but none of the other metabolites were correlated with *Bifidobacterium*. Similarly, reductions in 3-hydroxykynurenine were inversely correlated with changes in *Akkermansia* in the placebo group (*p* = 0.03; Table 2), but none of the other metabolites were correlated with *Akkermansia*.

## 4. Discussion

Elucidating the physiological benefits of prebiotics is a major objective for industry-led research. We report that consumption of RPS promoted increases in serum all-trans retinol and α-tocopherol, two fat-soluble antioxidant vitamins. CoQ10 levels were elevated in both treatment groups, while levels of uric acid and xanthine, as well as xanthine oxidase activity, were unaffected by treatment. Neither treatment influenced hippuric acid levels, but levels of 3-hydroxykynurenine were decreased in both treatment groups. Advanced glycation end products, which are markers oxidative stress, were not significantly affected by either treatment. Notably, increases in all-trans retinol were correlated with increases in *Akkermansia*, a genus of mucin-degrading gut bacteria associated with intestinal barrier health [34].

There are 5 categories of RS, with RPS belonging to the RS2 category. The RS2 group includes unmodified, naturally occurring starches that resist digestion due to their crystalline structure [35]. Not all unmodified starches contain substantial levels of RS, and RS2 ingredients from different sources can be biochemically distinct and produce different effects. For example, high amylose maize starch is almost entirely composed of linear amylose chains while RPS contains 80% branched amylopectin chains and 20% amylose [19]. These differences influence the composition of the gut microbiota and play a role in the prebiotic effects of these ingredients [18].

Most prebiotic ingredients are extracts that contain multiple biologically active components. Studies have demonstrated that antioxidant properties are discreet from prebiotic activities in these ingredients and are likely attributable to the antioxidant properties of polyphenols [36,37,38,39,40]. Resistant potato starch increased the absorption of antioxidant all-trans retinol and α-tocopherol. This is consistent with studies of other prebiotics showing enhanced nutrient absorption. For example, inulin and fructooligosaccharides enhance mineral absorption [41,42,43,44], as does high amylose maize starch [45]. Daily consumption of oligofructose-enriched inulin increases vitamin E but not vitamin A levels in children with celiac disease following a long-term gluten-free diet [46].

Increased serum levels of lipid-soluble vitamins in RPS-consuming participants could be due to improved intestinal barrier function [21]. Increases in the gut-barrier-associated genus *Akkermansia* were significantly correlated with increases in all-trans retinol and tended to be correlated with increases in α-tocopherol in RPS-consuming participants but not those consuming placebo. Other metabolites were not correlated with either *Akkermansia* or *Bifidobacterium* in either RPS- or placebo-consuming participants.

The R^2^ values suggest that *Akkermansia* changes explain 14.4% of the variation in all-trans retinol and 5.2% of the variation in α-tocopherol in RPS-consuming participants. The correlations between *Akkermansia* and the nutrients do not fully explain the increases in serum vitamin levels, suggesting that factors other than increased *Akkermansia* levels are influencing vitamin absorption. One possibility is that RPS increased the consumption of all-trans retinol, α-tocopherol, and/or CoQ10. Increases in these nutrients could not be explained by dietary supplements consumption because participants were prohibited from dietary supplement use during the trial [20]. Unfortunately, dietary levels of all-trans retinol and α-tocopherol were not captured during the study, precluding this analysis. Another possibility is that RPS consumption increased fat intake, which could increase the absorption of these lipid-soluble vitamins, as well as CoQ10 [47]. However, macronutrient intake was not affected by RPS or placebo consumption in this clinical trial [28]. Animal studies addressing the role of RPS in promoting lipid soluble vitamin absorption are required to elucidate the mechanisms of action.

The role of prebiotics influencing CoQ10 production has not been thoroughly explored. While the body can synthesize CoQ10 [7], this substance is a fat-soluble nutrient that can be obtained from the diet, especially from meat, fish, nuts, and some oils [8]. It is intriguing to speculate that RPS-dependent increases in serum CoQ10 levels are due to improved intestinal function and nutrient absorption. However, changes in CoQ10 levels were not correlated with changes in the gut-barrier-associated genus *Akkermansia* in the RPS group. Alternatively, RPS consumption may lead to increased endogenous CoQ10 production, reflecting a systemic response. Unfortunately, important CoQ10 precursors like 4-hydroxybenzoate and mevalonate [7] were not captured in our metabolomics panels, preventing analysis of CoQ10 synthesis. However, levels of the antioxidant uric acid and activity of xanthine oxidase, which produces uric acid, were unaffected. While we cannot determine whether RPS is promoting endogenous CoQ10 synthesis from our data, the absence of increased xanthine oxidase activity suggests that RPS does not stimulate an endogenous response to oxidative stress.

The CoQ10 increase in the placebo group further confounds the issue. The placebo is a fully digestible corn starch that does not have a prebiotic effect [27], but it is possible that increased carbohydrate consumption in both treatment arms contributed to this effect. Further work is needed to elucidate the mechanism(s) by which RPS and placebo influences CoQ10.

Diet-derived polyphenols and tryptophan metabolites can act as antioxidants due to their phenolic rings, which balance oxidative-reduction reactions in physiological systems [30,31]. Potatoes are the greatest vegetable source of phenolics in the American diet [13], though the phenolic content of RPS is unknown. It is possible that RPS consumption influenced either the consumption of polyphenol-containing fruits and vegetables, or that RPS influenced the digestion of polyphenols via the gut microbiome. However, we detected no effect on hippuric acid, a metabolite positively correlated with the intake of polyphenol-rich vegetable foods [30]. Our findings suggest that RPS does not contain polyphenols or that RPS does not affect dietary polyphenol metabolism.

Dietary interventions that shift the proportion of carbohydrates in the diet are known to influence protein fermentation in the gut [27,48]. Levels of tryptophan metabolites 3-hydroxyantranillic acid and kynurenine were not affected by treatment. However, levels of the neurotoxic tryptophan metabolite 3-hydroxykynurenine, an inducer of oxidative stress [49], decreased in both treatment groups. Decreases were not correlated with changes in *Bifidobacterium* or *Akkermansia* in the RPS group, but changes in 3-hydroxykynurenine were inversely correlated with changes in *Akkermansia* in the placebo group. While levels of 3-hydroxykynurenine decreased in the placebo group, *Akkermansia* levels did not increase [20], making it difficult to interpret the significance of these findings. Further work is needed to characterize how RPS influences protein fermentation and whether this influences biologically active amine absorption.

Despite increasing serum antioxidant levels, RPS supplementation had no effect on NƐ-(1-carboxymethyl)-L-lysine or NƐ-(1-carboxyethyl)-L-lysine, two markers of advanced end products of glycated proteins (AGEs) produced by oxidative stress [32,33]. In two separate studies, type 2 diabetic women consuming prebiotic resistant dextrin daily for 8 weeks experienced reductions in malonaldehyde, a marker of lipid peroxidation [50,51], and advanced glycation end products, including carboxymethyl lysine [51]. The present study was limited to 4 weeks, a period that is likely too short to detect changes in markers of oxidative stress. Studies examining the effect of diabetic self-management on AGE formation found that a duration of 4–6 months produced a significant reduction while less than three months did not [52], suggesting that future studies examining the effect of RPS on antioxidant activity be at least six months in length. In addition to this limitation, it is also possible that a higher dose may be required to see effects on glycation end products. This study population consisted of generally healthy adults, so studies examining RPS supplementation in a type 2 diabetic population might yield robust changes in these markers. Studies in these patients or other conditions involving elevated oxidative stress are warranted.

Curiously, participants in the placebo group experienced significant increases in CoQ10, reductions in 3-hydroxykynurenine, and trends towards increases in all-trans retinol, α-tocopherol, and kynurenine. The placebo consisted of fully digestible corn starch, which has no impact on the composition of the gut microbiome [27]. However, the placebo is digested and metabolized, so it is plausible that these changes are due to the metabolism of corn starch. Further work evaluating the effects of digestible corn starch on antioxidant-related metabolites are required to fully interpret the results of this study.

## 5. Conclusions

Dietary supplementation with RPS led to increases in serum all-trans retinol and α-tocopherol, which tended to be positively correlated with increases in the gut-barrier-associated genus *Akkermansia*. Increases in choline, all-trans retinol, and α-tocopherol absorption are consistent with improvements in intestinal barrier function in those consuming RPS [20]. However, imperfect correlations between microbial markers of barrier function and the absorption of these metabolites suggest that RPS promotes these increases via additional mechanisms. Studies in animal models of intestinal dysfunction will be required to elucidate the mechanisms of action by which RPS promotes serum increases in all-trans retinol, α-tocopherol, and CoQ10.

## 6. Patents

MSP Starch Products Inc. sister company of McPharma Biotech Inc. holds relevant patents US11058711B2, CA3024201A1, AU2017294806A1, and provisional patent application 63/712,780.

## Figures and Tables

**Table 1:** Correlations between changes in *Bifidobacterium* and *Akkermansia*, and changes in serum metabolites in RPS-consuming participants.

RPS	*Bifidobacterium*			*Akkermansia*		
	r	R^2^	*p*	r	R^2^	*p*
3-Hydroxyanthranillic Acid	0.078	0.006	0.6	0.188	0.035	0.2
3-Hydroxykynurenine	−0.188	0.035	0.2	0.142	0.020	0.3
All-trans Retinol	0.008	0.000	1.0	0.380	0.144	**0.008**
α-Tocopherol	0.030	0.001	0.8	0.227	0.052	0.1
CoQ10	0.128	0.016	0.4	0.120	0.014	0.4
Hippuric Acid	0.128	0.016	0.4	−0.115	0.013	0.4
Kynurenine	0.149	0.022	0.3	0.022	0.001	0.9
NƐ-(1-carboxymethyl)-L-lysine	0.055	0.003	0.7	−0.116	0.013	0.4
NƐ-(1-carboxyethyl)-L-lysine	−0.030	0.001	0.8	0.098	0.010	0.5
Uric Acid	0.038	0.001	0.8	0.133	0.018	0.4
Xanthine	0.046	0.002	0.8	−0.163	0.026	0.3

The *p* values of significant correlations are in bold.

**Table 2:** Correlations between changes in *Bifidobacterium* and *Akkermansia*, and changes in serum metabolites in Placebo-consuming participants.

Placebo	*Bifidobacterium*			*Akkermansia*		
	r	R^2^	*p*	r	R^2^	*p*
3-Hydroxyanthranillic Acid	−0.077	0.006	0.6	−0.249	0.062	0.09
3-Hydroxykynurenine	−0.046	0.002	0.8	−0.310	0.096	**0.03**
All-trans Retinol	−0.083	0.007	0.6	0.123	0.015	0.4
α-Tocopherol	−0.160	0.026	0.3	0.075	0.006	0.6
CoQ10	−0.325	0.106	**0.02**	0.037	0.001	0.8
Hippuric Acid	−0.011	0.000	0.9	0.203	0.041	0.2
Kynurenine	−0.134	0.018	0.4	−0.098	0.010	0.5
NƐ-(1-carboxymethyl)-L-lysine	−0.277	0.076	0.06	0.276	0.076	0.06
NƐ-(1-carboxyethyl)-L-lysine	−0.048	0.002	0.7	0.048	0.002	0.7
Uric Acid	−0.116	0.013	0.4	−0.019	0.000	0.9
Xanthine	0.096	0.009	0.5	0.086	0.007	0.6

The *p* values of significant correlations are in bold.

## Data Availability

The data presented in this study are available on request from the corresponding author, but the data are owned by MSP Starch Products Inc. and restrictions apply to the use of these data, including the execution of nondisclosure agreements and/or material transfer agreements.

## Abbreviations

The following abbreviations are used in this manuscript:

AGE	Advanced end products of glycated proteins
BMI	Body mass index
CoQ10	Coenzyme Q10
IS	Internal standard
LC-MS/MS	Liquid chromatography coupled to tandem mass spectrometry
QC	Quality control
ROS	Reactive oxygen species
RNS	Reactive nitrogen species
RPS	Resistant potato starch
RS	Resistant starch
SEM	Standard error of the mean
